# Improved quantification of absolute and differential pulmonary flow with highly-accelerated 4D-PC MRI

**DOI:** 10.1186/1532-429X-17-S1-Q93

**Published:** 2015-02-03

**Authors:** Albert Hsiao, Tashfeen Ekram, Marcus T Alley, Frandics Chan, Beverley Newman, Shreyas Vasanawala

**Affiliations:** 1 Department of Radiology, University of California, San Diego, San Diego, CA, USA; 2 Department of Radiology, Stanford University, Stanford, CA, USA

## Background

Conventional, planar phase-contrast (2D-PC) imaging is the gold standard for non-invasive measurement of blood flow, routinely used in the assessment of structural heart disease by MRI. Nevertheless, at many institutions, nuclear perfusion scintigraphy (NPS) remains necessary for confirmation of differential pulmonary perfusion, but requires an additional exam with radiation exposure, and in younger children prolongs cardiac anesthesia. Highly-accelerated 4D phase-contrast (4D-PC) MRI is an evolving technology that has potential to greatly simplify congenital heart MRI. We hypothesized that 4D-PC may be sufficient for quantification of differential pulmonary flow.

## Methods

With IRB approval and HIPAA-compliance, we retrospectively identified patients who underwent NPS as well as a cardiac MRI with 4D-PC from October 2011 through February 2014 without major surgery between exams. A total of 26 4D-PC examinations from 25 patients (15 male, 10 female) were identified. Aortic, main and branch pulmonary flow were quantified from 4D-PC. Pearson correlation and Bland-Altman analysis were used to analyze the quantitative consistency of 4D-PC data. The same analyses were then applied to compare differential pulmonary perfusion from 4D-PC against 2D-PC and NPS.

## Results

There was strong consistency between aortic flow and pulmonary flow measurements obtained at the pulmonary valve or as the sum of the branch pulmonary arteries (ρ=0.93, 0.90). Differential pulmonary flow measurements obtained from 4D-PC and NPS largely agreed (ρ=0.92), while correlation between 2D-PC and NPS was more modest (ρ=0.74). MRI and NPS were better matched among patients without substantial pulmonary regurgitation (RF<20%, n=15) whether obtained by 4D-PC (ρ=0.97) or 2D-PC (ρ=0.94). In contrast, the presence of substantial pulmonary regurgitation (RF≥20%, n=11) more severely impacted the accuracy of 2D-PC (ρ=0.47) than 4D-PC (ρ=0.89).

## Conclusions

Highly-accelerated 4D-PC is a viable alternative to NPS for evaluation of differential pulmonary perfusion, and has decreased sensitivity to turbulent flow that otherwise limits the accuracy of conventional 2D-PC. Highly-accelerated 4D-PC may not only help simplify congenital cardiac MRI, but may obviate the need for a separate nuclear scintigraphic examination to confirm differential pulmonary perfusion.

## Funding

Tashia and John Morgridge Faculty Scholar Fund. Lucas Foundation. NIH R01 EB009690, NIH P41 EB015891. General Electric, Sloan Research Fellowship, American Heart Association Grant #12BGIA9660006, NVIDIA.

**Figure 1:**
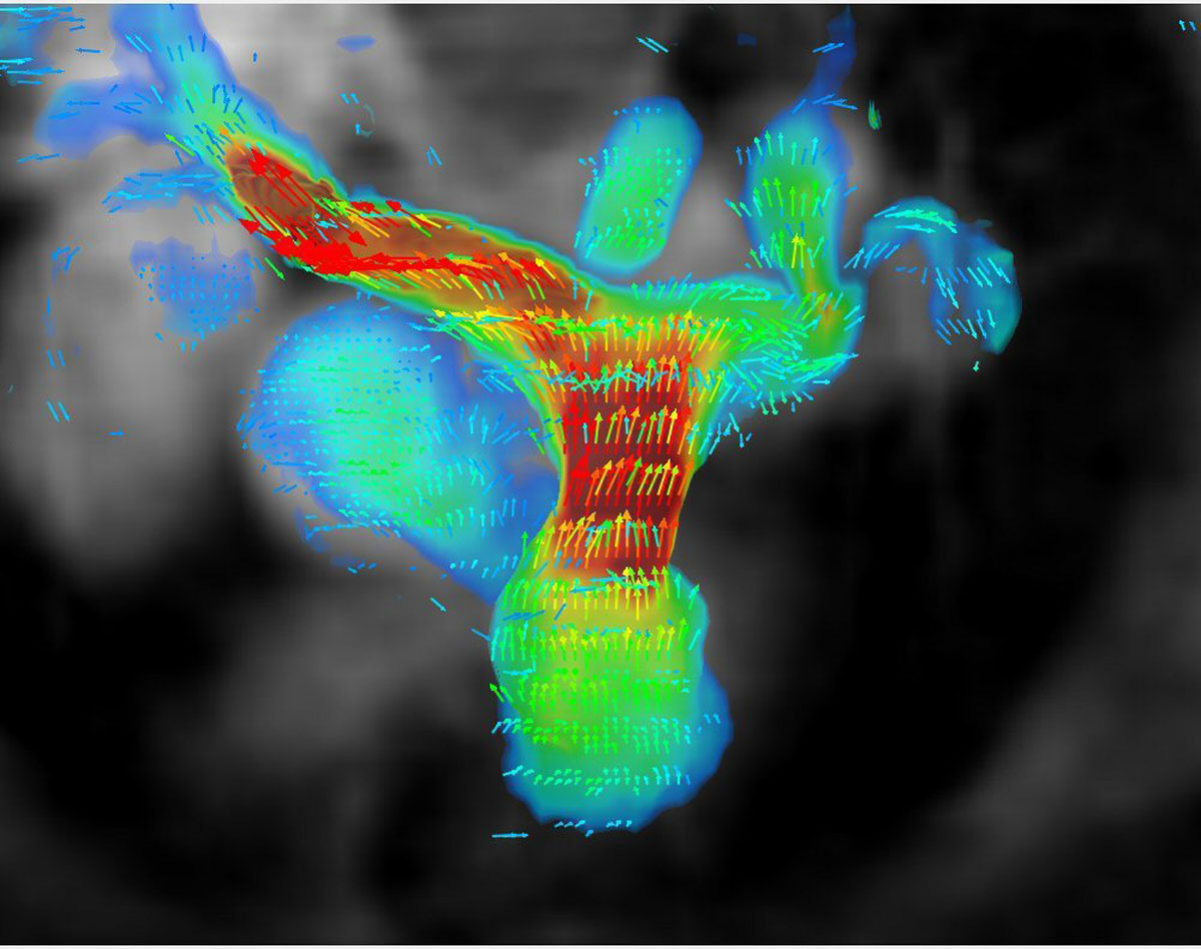
Cranial view of the pulmonary arteries in a patient with repaired Tetralogy of Fallot. Differential pulmonary perfusion by 4D-PC was measured at 58% to the right lung and 42% to the left lung, while regurgitant fraction at the pulmonary valve was measured at 39%. By nuclear scintigraphy, split perfusion was measured at 65% to the right lung and 35% to the left lung.

